# Association of diabetes with cardiovascular calcification and all-cause mortality in end-stage renal disease in the early stages of hemodialysis: a retrospective cohort study

**DOI:** 10.1186/s12933-024-02318-8

**Published:** 2024-07-18

**Authors:** Qingxian Li, Peishan Li, Zigan Xu, ZeYuan Lu, Chuan Yang, Jie Ning

**Affiliations:** 1 Department of Endocrinology, Shenzhen Longhua District Central Hospital, Shenzhen, 518110 China; 2 Department of Endocrinology, Sun Yat-Sen Memorial Hospital, Sun Yat-Sen University, Guangzhou, 510120 China; 3 Department of Nephrology, Shenzhen Longhua District Central Hospital, Shenzhen, 518110 China; 4 Department of Endocrinology, The Eighth Affiliated Hospital of Sun Yat-Sen University, Shenzhen, 518033 China

**Keywords:** Diabetes, The early stages of hemodialysis, Cardiovascular calcification, Mortality, Prediction

## Abstract

**Background:**

The main goal of this study was to examine how diabetes, cardiovascular calcification characteristics and other risk factors affect mortality in end-stage renal disease (ESRD) patients in the early stages of hemodialysis.

**Methods:**

A total of 285 ESRD patients in the early stages of hemodialysis were enrolled in this research, including 101 patients with diabetes. Survival time was monitored, and general data, biochemical results, cardiac ultrasound calcification of valvular tissue, and thoracic CT calcification of the coronary artery and thoracic aorta were recorded. Subgroup analysis and logistic regression were applied to investigate the association between diabetes and calcification. Cox regression analysis and survival between calcification, diabetes, and all-cause mortality. Additionally, the nomogram model was used to estimate the probability of survival for these individuals, and its performance was evaluated using risk stratification, receiver operating characteristic, decision, and calibration curves.

**Results:**

Cardiovascular calcification was found in 81.2% of diabetic patients (82/101) and 33.7% of nondiabetic patients (62/184). Diabetic patients had lower phosphorus, calcium, calcium-phosphorus product, plasma PTH levels and lower albumin levels (p < 0.001). People with diabetes were more likely to have calcification than people without diabetes (OR 5.66, 95% CI 1.96–16.36; p < 0.001). The overall mortality rate was 14.7% (42/285). The risk of death was notably greater in patients with both diabetes and calcification (29.27%, 24/82). Diabetes and calcification, along with other factors, collectively predict the risk of death in these patients. The nomogram model demonstrated excellent discriminatory power (area under the curve (AUC) = 0.975 at 5 years), outstanding calibration at low to high-risk levels and provided the greatest net benefit across a wide range of clinical decision thresholds.

**Conclusions:**

In patients with ESRD during the early period of haemodialysis, diabetes significantly increases the risk of cardiovascular calcification, particularly multisite calcification, which is correlated with a higher mortality rate. The risk scores and nomograms developed in this study can assist clinicians in predicting the risk of death and providing individualised treatment plans to lower mortality rates in the early stages of hemodialysis.

**Supplementary Information:**

The online version contains supplementary material available at 10.1186/s12933-024-02318-8.

## Introduction

Globally, chronic kidney disease (CKD) has emerged as a substantial health burden [1]. CKD stage 5 represents the most severe phase of the condition. The high prevalence of vascular calcification among patients with end-stage renal disease (ESRD) undergoing hemodialysis (HD) is well-documented, highlighting their elevated risk for a variety of cardiovascular complications, including coronary artery disease, heart failure, arrhythmias,and sudden cardiogenic death [2, 3]. Cardiovascular complications rank first in terms of mortality among patients with renal failure who are regularly receiving maintenance HD treatment [2]. The global incidence of cardiovascular disease-related fatalities notably increased by 21.1% from 2007 to 2017 [4]. Furthermore, diabetes-related CKD accounts for an 18.9% increase in fatalities [4].

The incidence of cardiovascular calcification (CVC) is considerably greater in patients with CKD, particularly those on dialysis for ESRD, and its incidence and severity are independent predictors of CVC and mortality [5, 6]. The Kidney Disease Improving Global Prognosis (KDIGO) 2017 guidelines recommend that patients with CKD stages 3a to 5 who develop vascular or cardiac valve calcification have the highest risk of cardiovascular events [7]. Among the various risk factors for vascular calcification, diabetes is recognized as a significant risk factor, in addition to traditional risk factors such as advanced age, hypertension, and dyslipidaemia [2]. Patients with diabetes mellitus (DM) are more susceptible to progressive calcification and arteriosclerosis, which in turn elevates the risk of cardiovascular events such as hypertension, stroke, myocardial infarction, and amputation, as well as the risk of premature mortality [8–12].

Currently, early intervention measures are critical for patients in the early stages of HD due to their elevated risk of calcification and mortality compared to the general population. The literature predominantly focuses on long-term HD patients, with limited research addressing cardiovascular complication risk factors in newly initiating HD patients with diabetes. This gap underscores the need for further studies on the impact of diabetes and calcification on mortality in this specific population. Therefore, we conducted a comparative analysis of the characteristics of CVC in diabetic and nondiabetic patients in the early stages of haemodialysis, which may help further clarify their risk factors, investigate their survival prognosis, explore possibilities for mitigating CVC progression and improve clinical outcomes through optimised disease management.

## Methods

### Study design and participants

This study retrospectively analysed the clinical data of patients who received early-stage haemodialysis, defined as the time from the initiation of haemodialysis treatment to the end of the first two years, at the Shenzhen Longhua District Central Hospital from 2015 to 2022. Patients aged 18 or older with less than two years of HD and no sex or ethnicity restrictions were included. We excluded patients who were pregnant, breastfeeding, suffering from rheumatic heart disease or congenital heart disease, who had undergone surgical or interventional treatment for valvular disease, post parathyroidectomy, arrhythmias, multiple myeloma, amyloidosis, chronic liver disease, systemic lupus erythematosus, potential malignant tumours, severe infections or other conditions causing renal insufficiency, and who had considerable clinical data deficiencies. The study ultimately enrolled 285 patients, of whom 101 (35.4%) had diabetes, 92 (91.1%) had diabetic nephropathy, and 9 (8.9%) had CKD combined with diabetes; the non-diabetic group (64.6%) comprised 184 patients, of whom 99 (53.8%) had chronic nephritis, 35 (19.0%) had hypertensive nephropathy, and 50 (27.2%) had other renal diseases (Supplementary file 2: Figure S1).

### Data collection and definitions

The demographic and clinical variables included age, sex, medical history of diabetes, coronary heart disease (CHD) and stroke, smoking history, body mass index (BMI), 24-h urine volume, duration of renal failure, and hypertension duration. Diabetes was diagnosed based on the World Health Organization’s criteria (1999). The serological indices collected were haemoglobin (Hb), HbA1c, albumin (ALB), total cholesterol (TC), triglycerides (TG), low-density lipoprotein cholesterol (LDL-c), high-density lipoprotein cholesterol (HDL-c), serum creatinine (Scr), urea (UA), intact parathyroid hormone (iPTH), phosphorus (Pi), and calcium (Ca). For patients with an ALB concentration less than 40 g/L, corrected calcium was calculated using the following formula: corrected calcium (mmol/L) = measured calcium − 0.02 × (ALB − 40). The calcium-phosphorus ratio was calculated as Ca (mmol/L) × Pi (mmol/L) × 12.4. Given the advanced renal impairment in HD patients, their estimated glomerular filtration rate (eGFR) was not calculated. The blood samples were collected in the morning, in a fasting state, before the first dialysis session. All patients underwent a standardised HD protocol of 12 h per week, typically three 4-h sessions per week. This protocol ensured adequate dialysis treatment and adhered to established guidelines for HD duration and frequency.

The above information was collected and validated by two seasoned clinicians, and all data requests were approved and approved by the Shenzhen Longhua District Central Hospital Ethics Committee.

### Cardiovascular calcification (CVC) and mortality outcomes

Cardiac colour Doppler ultrasound and chest computed tomography (CT) scans were performed within the first two weeks of initiating HD. Considering the differences in the sites of calcification, we classified the calcification into three regions: heart valve calcification, coronary artery calcification, and thoracic aorta calcification. The criteria for determining heart valve calcification were mainly based on cardiac Doppler ultrasound, which was determined by observing one or more echogenic solid refractions ≥ 1 mm in the aortic valve, mitral valve, or annulus. The calcification of the coronary arteries and thoracic aorta was assessed using CT scanning and a radiologist confirmed the findings. Calcification in any of these areas was considered positive for CVC.

All-cause mortality was the primary endpoint, defined as the total number of deaths from any cause with participants’ survival tracked from January 1, 2015, to December 31, 2022, using hospital records or family-provided death information. All natural death events were included.

### Statistical analysis

The study population was divided into diabetic and nondiabetic groups. Continuous variables are reported as the mean ± SD. For normally distributed data, *t-tests* were used to compare the groups, while no normally distributed data were analysed using the *Wilcoxon rank-sum test*. Categorical data are expressed as percentages and were compared using the *chi-square* or *Cochran–Mantel–Haenszel (CMH)* test.

To examine the association between diabetes and calcification, three logistic regression models were sequentially built to test the robustness of the results. Model 1 was unadjusted (only diabetes). Model 2 was adjusted for age and sex, and Model 3 was further adjusted for CHD/stroke, ALB, HbA1c, Scr, BUN, Ca, Pi, iPTH, TC, TG, LDL-C, and HDL-C. Subgroup analyses were performed based on sex (male, female), age (< 50, ≥ 50), albumin (< 35 g/L, ≥ 35 g/L), creatinine (< 707 μmol/L, ≥ 707 μmol/L), 24-h urine volume (< 1000 ml, ≥ 1000 ml), Ca (< 2 mmol/L, ≥ 2 mmol/L), Pi (< 1.8 mmol/L, ≥ 1.8 mmol/L), and LDL-C (< 1.8 mmol/L). Multiple interactions were assessed to evaluate differences between subgroups.

A multivariate-adjusted Cox regression model was used to assess the impact of calcification and diabetes on mortality risk. Participants were categorized into four groups based on the number of calcification sites: 0, 1, 2, and 3, analyzed as both continuous and categorical variables (with the 0-site group as the reference). Additionally, subjects were divided into four groups based on the presence of calcifications and diabetes: non-DM & non-CA, non-DM & CA, DM & non-CA, and DM & CA (with the non-DM & non-CA group as the reference). Cox regression models, Kaplan–Meier (K–M) survival plots, and *log-rank tests* were employed to compare survival rates across these groups.

A mortality risk model was constructed using significant clinical features from Table 1. The model was constructed using features with nonzero coefficients selected through LASSO regression with 100 bootstrap iterations. The selected indicators were assessed for multicollinearity using Spearman’s rank correlation and the variance inflation factor (VIF). Nomograms and risk-linkage plots were created to illustrate how each variable predicted mortality risk. Patients' total scores were calculated, and the study population was divided into high- and low-risk groups using a determined cut-off value. K‒M survival curves were used to compare survival times between these groups to validate the ability of the nomogram model to predict survival. The model’s performance was further assessed using the time-dependent area under the curve (AUC) curves to compare different models over time. The DeLong test was used to evaluate the differences in the AUC. Additionally, calibration and decision curves were employed to further evaluate the model’s performance.

Data analysis and visualisation were conducted using R (version 4.4.0, Windows 11) and Microsoft PowerPoint 2021. Key packages included *forestploter* and *ggplot2* for forest plots, *ggrisk* for risk-linkage maps, *adjusted curves* for survival curves, *glmnet* for LASSO regression, *corrplot* for heatmaps, *DynNom* for nomograms, and *survminer* for determining cut-off values. The *timeROC* package was used to calculate time-dependent ROC curves, *ggDCA was used* for decision curve analysis, and *plotCalibration* was used for calibration curves. Statistical significance was defined as *p* < *0.05*.

## Results

### Baseline characteristics

Table 1 compares the baseline characteristics of 285 haemodialysis patients sorted into diabetes (101, 35.4%) and nondiabetes (184, 34.7%) groups. The study participants included 195 males (68.4%). The average population age was 50.26 ± 15.00 years. The population all-cause mortality rate was 14.7% (42/285). Compared to nondiabetic patients, diabetic patients were older and exhibited lower blood phosphorus, calcium-phosphorus products, albumin, and iPTH levels (p < 0.05, Table 1). Patients with diabetes had significantly greater rates of heart valve, coronary artery, and aortic calcification (p < 0.001, Table 1), with 32.7% vs. 9.2%, 65.3% vs. 15.2%, and 72.3% vs. 32.1%, respectively. Furthermore, patients with diabetes had greater multisite calcification than nondiabetic patients (p < 0.001, Table 1). The all-cause mortality rate was greater in diabetic individuals (26.7% vs. 8.2%, p < 0.001; Table 1).

**Table 1:** Baseline characteristics of participants between diabetes and non-diabetes

		**Total (n=285)**	**diabetes (n=101)**	**non-diabetes (n=184)**	***p-value***
Age (years)		50.26±15.00	58.23±12.65	45.89±14.41	<0.001
Sex (%)	male	195 (68.4)	68 (67.3)	127 (69.0)	0.768
Smoke (%)	Yes	30 (10.6)	15 (15.0)	15 (8.2)	0.078
	No	253 (89.4)	85 (85.0)	167 (91.8)	
CHD/stroke	Yes	55 (19.5)	40 (39.6)	15 (8.2)	<0.001
	No	227 (80.5)	61 (60.4)	169 (91.9)	
BMI		23.05±4.26	22.86±3.29	23.19±4.85	0.645
ALB (g/L)		35.58±5.50	32.87±5.21	37.06±5.09	<0.001
duration of renal failure (months)		11.78±7.49	12.42±6.01	11.25±8.52	0.013
Hypertension time (years)		6.04±7.21	6.41±6.83	5.79±7.46	0.074
24h urine (ml)		1054.41±403.17	994.24±392.39	1089.93±406.57	0.062
HbA1c (%)		5.87±1.32	6.54±1.703	5.44±0.71	<0.001
Hb (g/L)		84.12±20.52	84.51±17.34	83.9±22.11	0.465
TC (mmol/L)		4.32±1.40	4.54±1.80	4.19±1.10	0.370
TG (mmol/L)		1.62±1.06	1.68±0.95	1.59±1.12	0.178
LDL (mmol/L)		2.14±0.78	2.2±0.89	2.11±0.70	0.430
HDL (mmol/L)		1.12±0.33	1.14±0.36	1.11±0.32	0.420
Scr (mg/dL)		1003.91±473.14	691.79±271.77	1175.23±473.06	<0.001
Carbamide (mmol/L)		29.74±12.6	25.11±10.83	32.29±12.81	<0.001
Ca (mmol/L)		2.02±0.29	2.09±0.26	1.98±0.30	0.001
Pi (mmol/L)		2.22±0.67	1.87±0.55	2.42±0.66	<0.001
Ca-Pi product (mg/dl)		54.73±16.30	48.03±14.01	58.4±16.33	<0.001
iPTH (pg/ml)		28.70±17.41	23.44±16.85	31.44±17.11	<0.001
Calcification	Yes	144 (50.5)	82 (81.2)	62 (33.7)	<0.001
	No	141 (49.5)	19 (18.8)	122 (66.3)	
Calcification number	0	141 (49.5)	19 (18.8)	122 (66.3)	<0.001
	1	54 (19.0)	21 (20.8)	33 (17.9)	
	2	47 (16.5)	31 (30.7)	16 (8.7)	
	3	43 (15.1)	30 (29.7)	13 (7.1)	
Heart Valves Calcification	Yes	51 (17.9)	34 (33.7)	17 (9.2)	<0.001
	No	234 (82.1)	67 (66.3)	167 (90.8)	
Coronary Artery Calcification	Yes	94 (33.0)	66 (65.3)	28 (15.2)	<0.001
	No	191 (67.0)	35 (34.7)	156 (84.8)	
Aortic Calcification	Yes	132 (46.3)	73 (72.3)	59 (32.1)	<0.001
	No	153 (53.7)	28 (27.7)	125 (67.9)	
All-cause motality		42 (14.7)	27 (26.7)	15 (8.2)	<0.001

Data are shown as mean ± SD or n (%)

*CHD* coronary heart disease. *BMI* body mass index. *SBP* systolic blood pressure. *DBP* diastolic blood pressure. *ALB* albumin. *HbA1c* blood glycosylated hemoglobin. *Pi* phosphate. *Ca* corrected calcium. *Hb* hemoglobin. *Scr* serum creatinine. *TC* total cholesterol. *TG* triglyceride. *LDL-C* low-density lipoprotein cholesterol. *HDL* high-density lipoprotein cholesterol. *eGFR* estimate glomerular filtration rate.

### Impact of diabetes on calcification

**Fig. 1:**
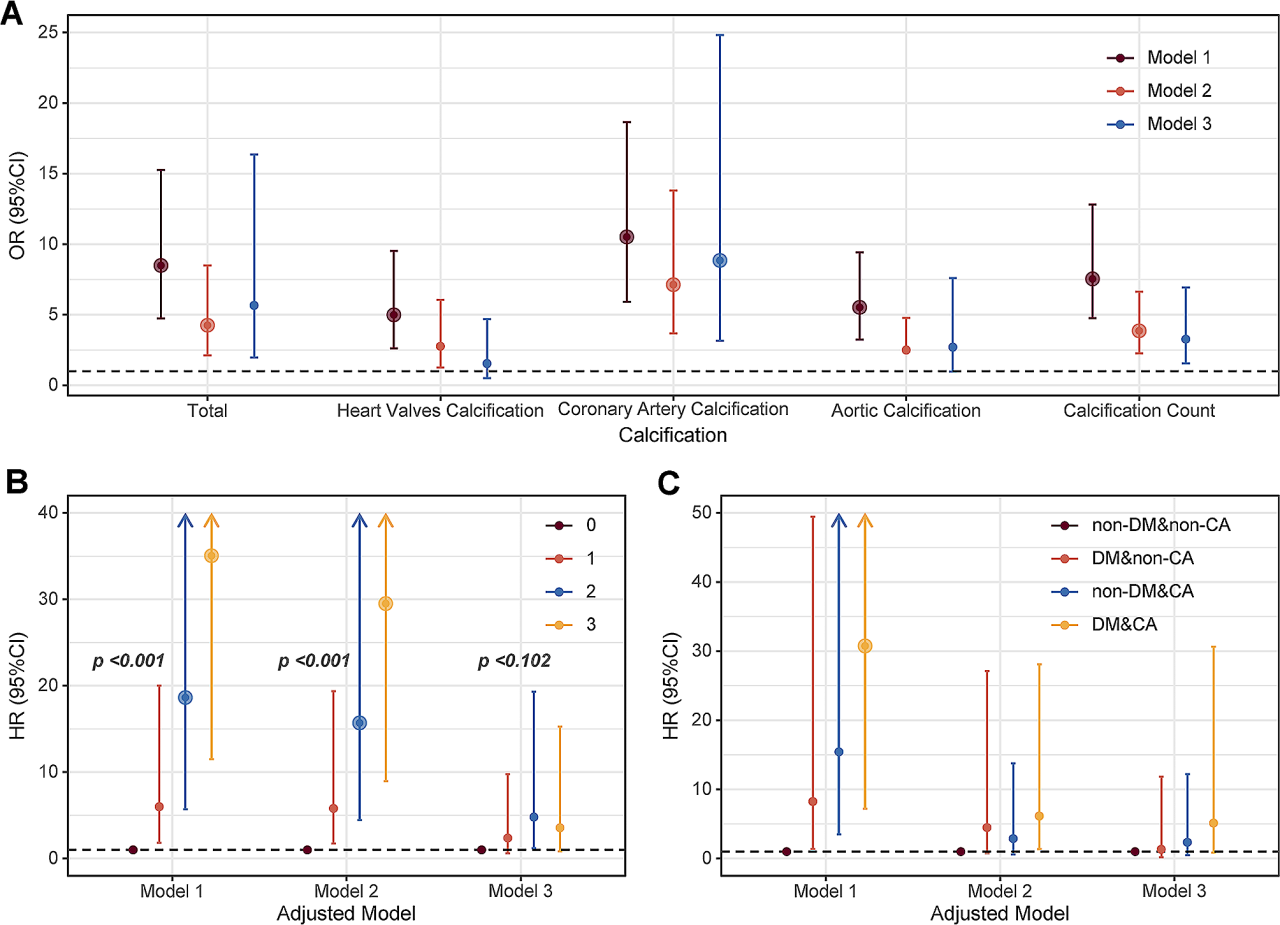
Forest plot of the influence of diabetes on calcification (**A**), the effect of calcification counts (**B**), and calcification and diabetes on Mortality (**C**). Adjustment of Model as shown in Tables 2 and 3. *p*, *p trend*. *DM* diabetes, *CA* Calcification, *OR* odds radio, *HR* hazard ratio, *CI* confidence interval

**Fig. 2:**
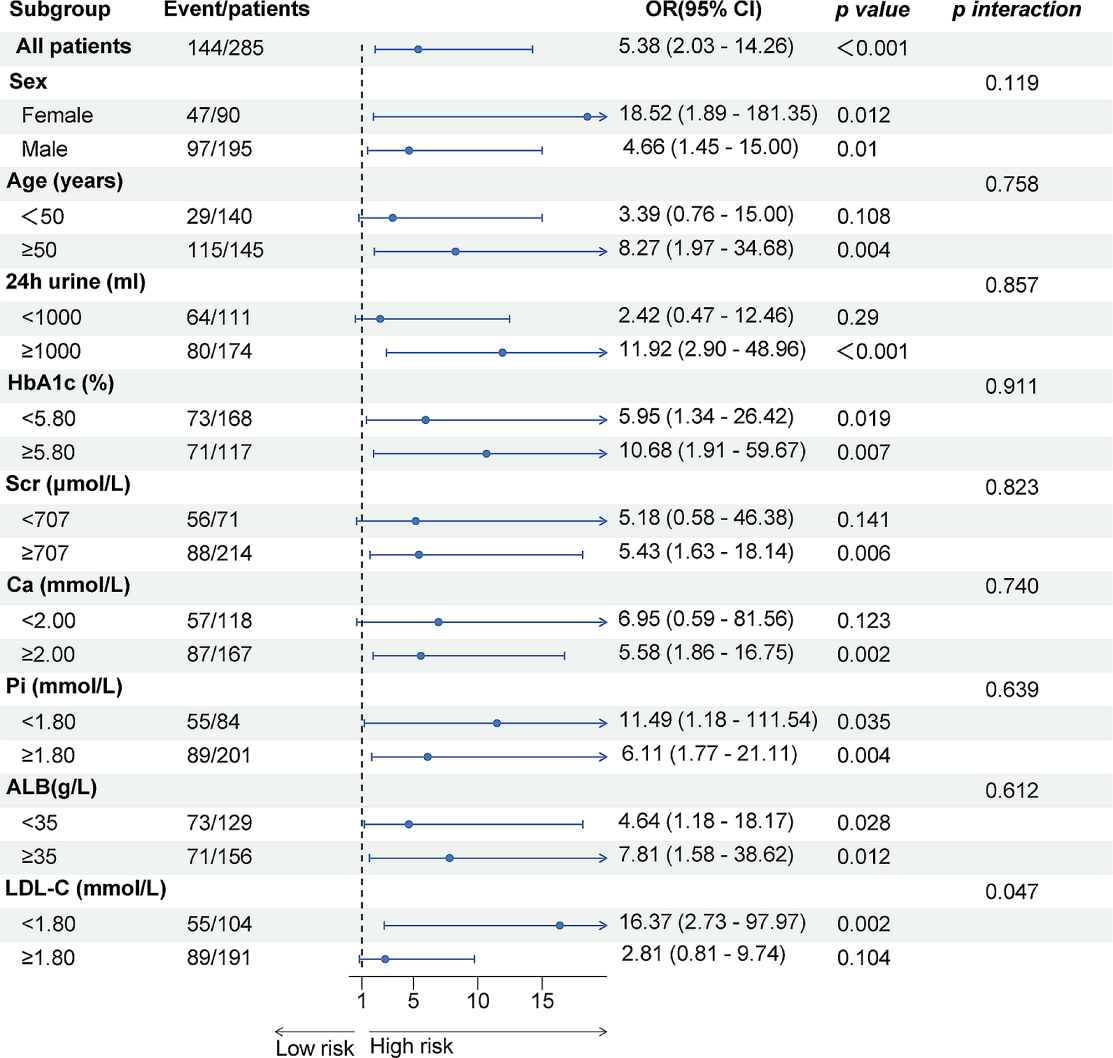
Subgroup analysis of the effect of diabetes on cardiovascular calcification in hemodialysis patients. The above models are adjusted by sex, age, CHD/stroke, Scr, BUN, Pi, iPTH, HbA1c. *OR* odds radio, *CI* confidence interval

Diabetes was a risk factor for both CVC (OR: 8.49, 95% CI: 4.73–15.25, p < 0.001, Table 2) and multisite calcification (OR: 7.54, 95% CI: 4.75–12.81, p < 0.001, Table 2), according to the results (without adjusting for confounding variables). After adjusting for potential confounders, diabetes remained a risk factor (CVC: OR 5.66, 95% CI 1.96–16.36, p < 0.001; calcification count: OR 3.27, 95% CI 1.56–6.92, p = 0.001, Table 2). According to all the models, diabetes was strongly associated with coronary calcification (OR 8.85, 95% CI 3.16–24.83; p < 0.001, Table 2). The correlation between diabetes factors and different calcification sites, analyzed using various models, is also illustrated in the longitudinal forest plot (Fig. 1A). In addition, subgroup analyses of different groups were performed (Fig. 2). Diabetes was significantly associated with CVC in most of the subgroups (p < 0.05, Fig. 2), except for the subgroups of age < 50 y, urine (24 h) < 1000 ml, Scr < 707 μmol/L, Ca < 2.0 mmol/L, and LDL-C ≥ 1.80 mmol/L. Further interaction analyses revealed no significant multiplicative interactions between diabetes and most of the subgroups (p interaction > 0.05, Fig. 2), except for LDL-C (p interaction 0.047, Fig. 2).

**Table 2:** The influence of diabetes on calcification by logistic regression

Event/N	Model 1		Model 2		Model 3	
	OR (95% CI)	p-value	OR (95% CI)	p-value	OR (95% CI)	p-value
Calcification (Yes, No)						
144/285	8.49 (4.73–15.25)	< 0.001	4.25 (2.12–8.50)	< 0.001	5.66 (1.96–16.36)	< 0.001
Calcification location						
Heart valves calcification (Yes, No)						
51/280	4.99 (2.61–9.53)	< 0.001	2.76 (1.26–6.06)	0.011	1.54 (0.51–4.69)	0.448
Coronary artery calcification (Yes, No)						
94/273	10.51 (5.92–18.66)	< 0.001	7.13 (3.68–13.80)	< 0.001	8.85 (3.16–24.83)	< 0.001
Aortic calcification (Yes, No)						
132/273	5.52 (3.24–9.43)	< 0.001	2.50 (2.40–4.77)	0.005	2.70 (0.96–7.60)	0.060
Calcification count (0, 1, 2, 3)						
	7.54 (4.76–12.81)	< 0.001	3.86 (2.27–6.63)	< 0.001	3.27 (1.56–6.92)	0.001

Model 1, only diabetes. Model 2, Adjusted by model 1 + age + sex. Model 3, Adjusted by model 2 + CHD/stroke + ALB + HbA1c + Scr + BUN + Ca + Pi + iPTH + TC + TG + LDL-C + HDL-C

*OR* odds radio, *CI* confidence interval

### Impact of diabetes and calcification on death

**Fig. 3:**
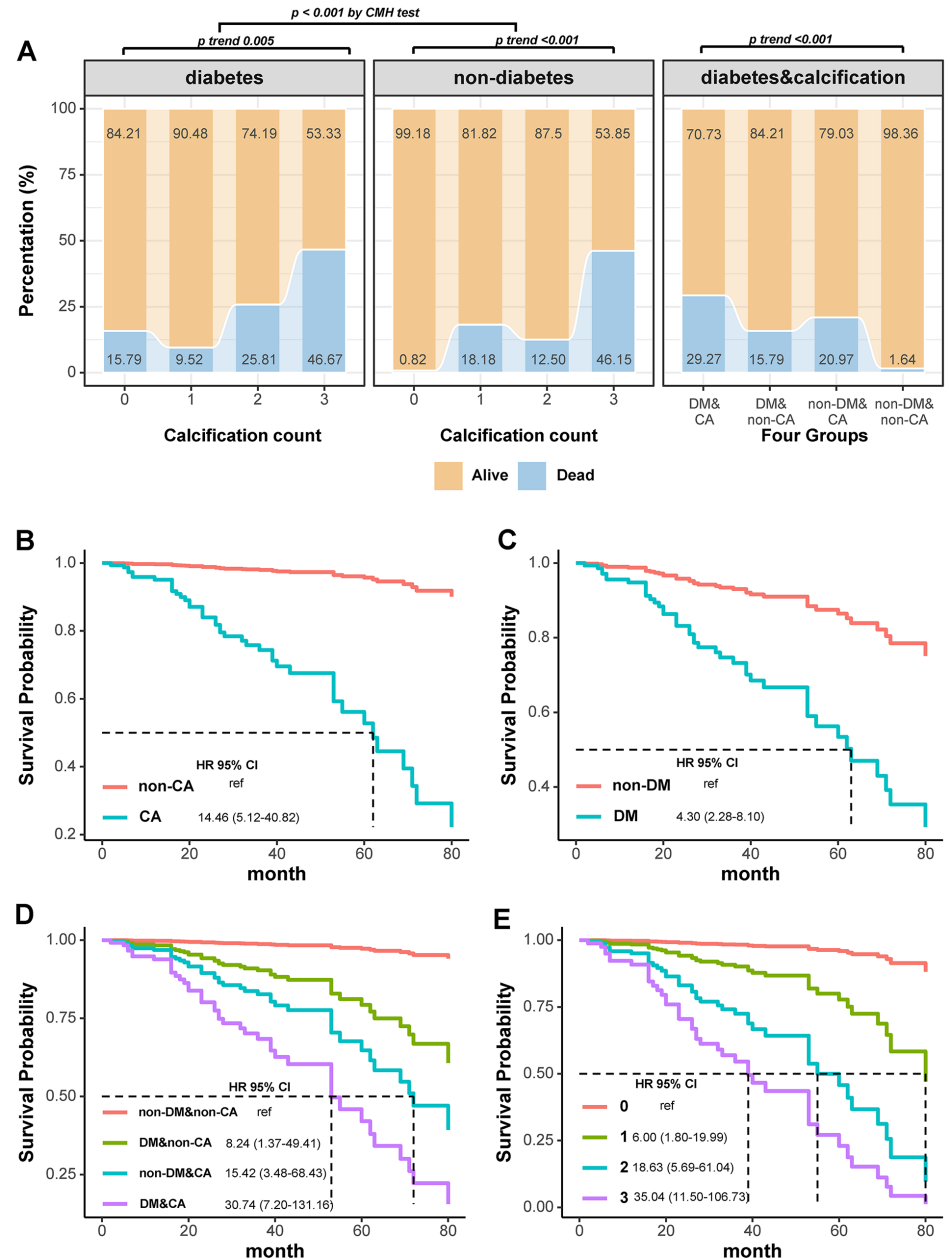
Mortality differences of calcification counts in diabetes or not and different groups (**A**). Survival curves between calcification and non-calcification group (**B**), diabetes and non-diabetes (**C**) and different groups (**D**). Survival curves among different counts of calcification (**G**). *DM* diabetes, *CA* calcification count, *HR* hazard ratio, *CI* confidence interval, *ref* reference

Overall mortality rates in patients with DM were greater than non-DM patients (26.7% vs. 8.2%, p < 0.001, Table 1). Mortality rates increased with the number of calcification sites (p trend < 0.001, Fig. 3A), and this difference persisted among patients with diabetes (p < 0.001 by CMH test, Fig. 3A). The mortality rates for the non-DM&non-CA, DM&non-CA, non-DM&CA, and DM&CA groups were significantly different at 0.82% (2/122), 15.79% (3/19), 20.97% (13/62), and 29.27% (24/82), respectively (p < 0.001, Fig. 3A & Table 3). According to Model 1, DM and CA patients had a significantly greater mortality risk than non-DM and non-CA patients (HR: 30.74, 95% CI: 7.20–131.16, p = 0.012, Table 3), followed by non-DM and CA patients (HR: 15.42, 95% CI: 3.48–68.43, p = 0.001, Table 3) and DM and non-CA patients (HR: 8.24, 95% CI: 1.37–49.41, p < 0.001, Table 3). After adjusting for age and sex in Model 2, only the DM and CA groups remained significantly associated with increased mortality (HR: 6.15, 95% CI: 1.35–28.10, p = 0.019, Table 3). Further adjustment in Model 3 reduced the significance of the associations, with the DM and CA groups showing a borderline significance (HR: 5.13,95% CI: 0.86–30.63, p = 0.0737, Table 3). The aforementioned multi-model adjustments are further visualized through the longitudinal forest plots (Fig. 1B-C). K‒M survival curves indicated lower survival rates for CA patients (HR: 14.46, 95% CI: 5.12–40.82, p < 0.001, Fig. 3B), DM patients (HR: 4.30, 95% CI: 2.28–8.10, p < 0.001, Fig. 3C), and the DM & CA group, which had the worst survival outcome (HR: 30.74, 95% CI: 7.20–131.16, p < 0.001, Fig. 3D), followed by the non-DM&CA and DM & non-CA groups.

**Table 3:** The influence of Calcification and Diabetes on Mortality by Cox regression

Event/N		Model 1		Model 2		Model 3	
		HR (95% CI)	p-value	HR (95% CI)	p-value	HR (95% CI)	p-value
Continous		2.93 (2.20–3.91)	< 0.001	2.74 (1.98–3.78)	< 0.001	1.41 (0.93–2.13	0.102
0 (n = 141)	4/141	Reference	–	Reference	–	Reference	–
1 (n = 54)	8/54	6.00 (1.80–19.99)	0.004	5.79 (1.73–19.36)	0.004	2.37 (0.58–9.76)	0.231
2 (n = 47)	10/47	18.63 (5.69–61.04)	< 0.001	15.69 (4.44–55.46)	< 0.001	4.80 (1.19–19.31)	0.027
3 (n = 43)	20/43	35.04 (11.50–106.73)	< 0.001	29.50 (8.95–97.25)	< 0.001	3.55 (0.82–15.25)	0.089
p for trend		< 0.001		< 0.001		0.102	
Diabetes (Yes, No) &Calcification (Yes, No)							
Group		Model 1		Model 2		Model 3	
non-DM & non-CA (n = 122)	2/122	Reference	–	Reference	–	Reference	–
DM & non-CA (n = 19)	3/19	8.24 (1.37–49.41)	0.021	4.47 (0.74–27.08)	0.103	1.33 (0.15–11.82)	0.799
non-DM & CA (n = 62)	13/62	15.42 (3.48–68.43)	0.001	2.88 (0.60–13.76)	0.184	2.34 (0.45–12.18)	0.313
DM & CA (n = 82)	24/82	30.74 (7.20–131.16)	< 0.001	6.15 (1.35–28.10)	0.019	5.13 (0.86–30.63)	0.073

The influence of calcification counts on mortality: Model 1 only calcification count. Model 2 adjusted by model 1 + diabetes. Model 3 adjusted by model 2 + age + sex + CHD/stroke + ALB + HbA1c + Scr + BUN + Ca + Pi + iPTH + TC + TG + LDL-C + HDL-C

The influence of calcification and diabetes on mortality: Model 1 only 4 groups. Model 2 adjusted by model 1 + age + sex. Model 3 adjusted by model 2 + CHD/stroke + ALB + HbA1c + Scr + BUN + Ca + Pi + iPTH + TC + TG + LDL-C + HDL-C

*OR* odds radio, *HR* hazard ratio, *CI* confidence interval

### Construction and validation of the predictive nomogram 

**Fig. 4:**
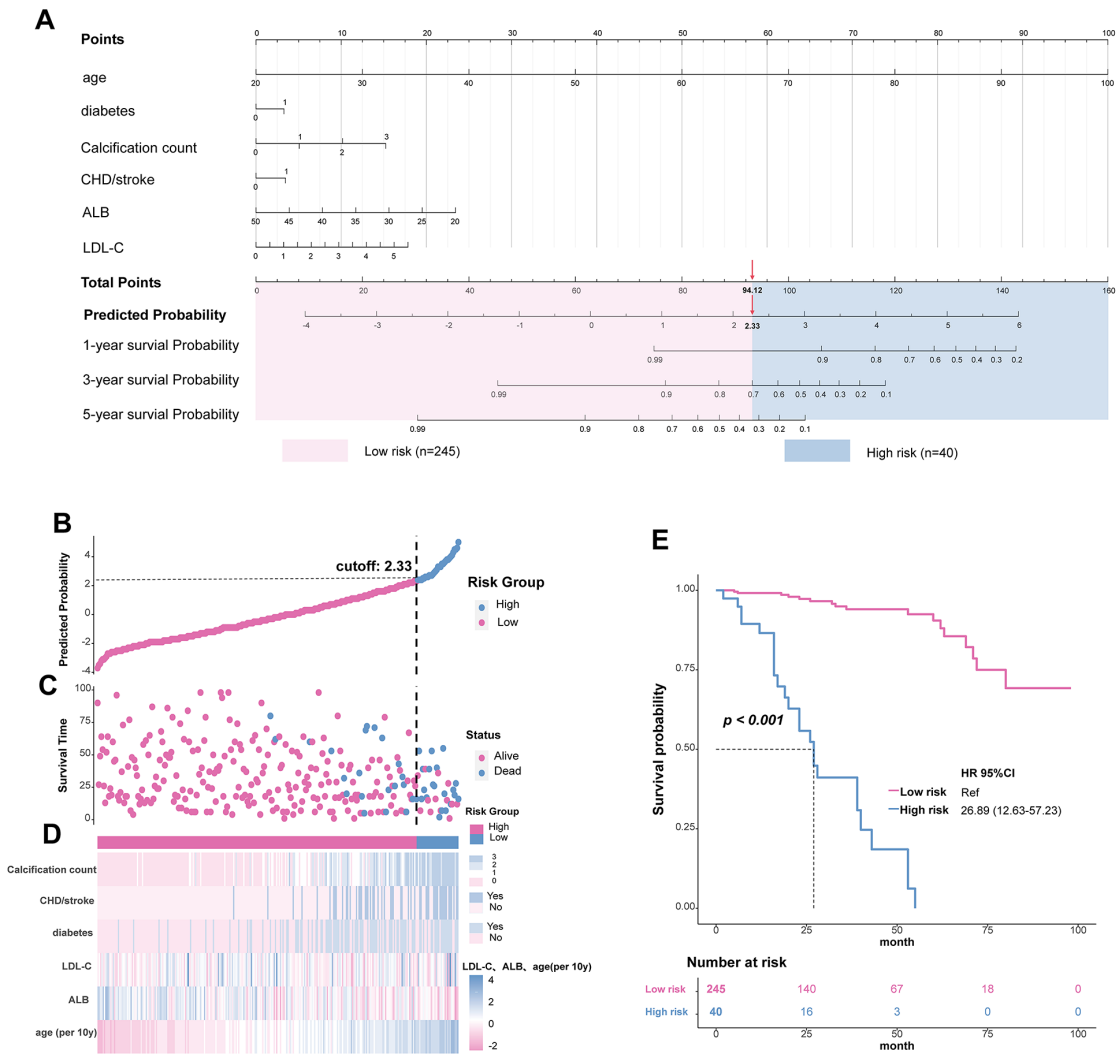
Development of a nomogram for the all-cause mortality in 5 years (**A**). Predictive value of the risk score model (**B** and **C**). Expression of the clustered heat map for the six variables in the model (**D**). Survival curves for high and low risk groups (**E**). The points calculated by the nomogram were cut off at the optimal value, as determined by the significance of the plots (94.12), dividing them into high-risk (25/40, 62.5%) and low-risk (17/245, 6.9%) groups

**Fig. 5:**
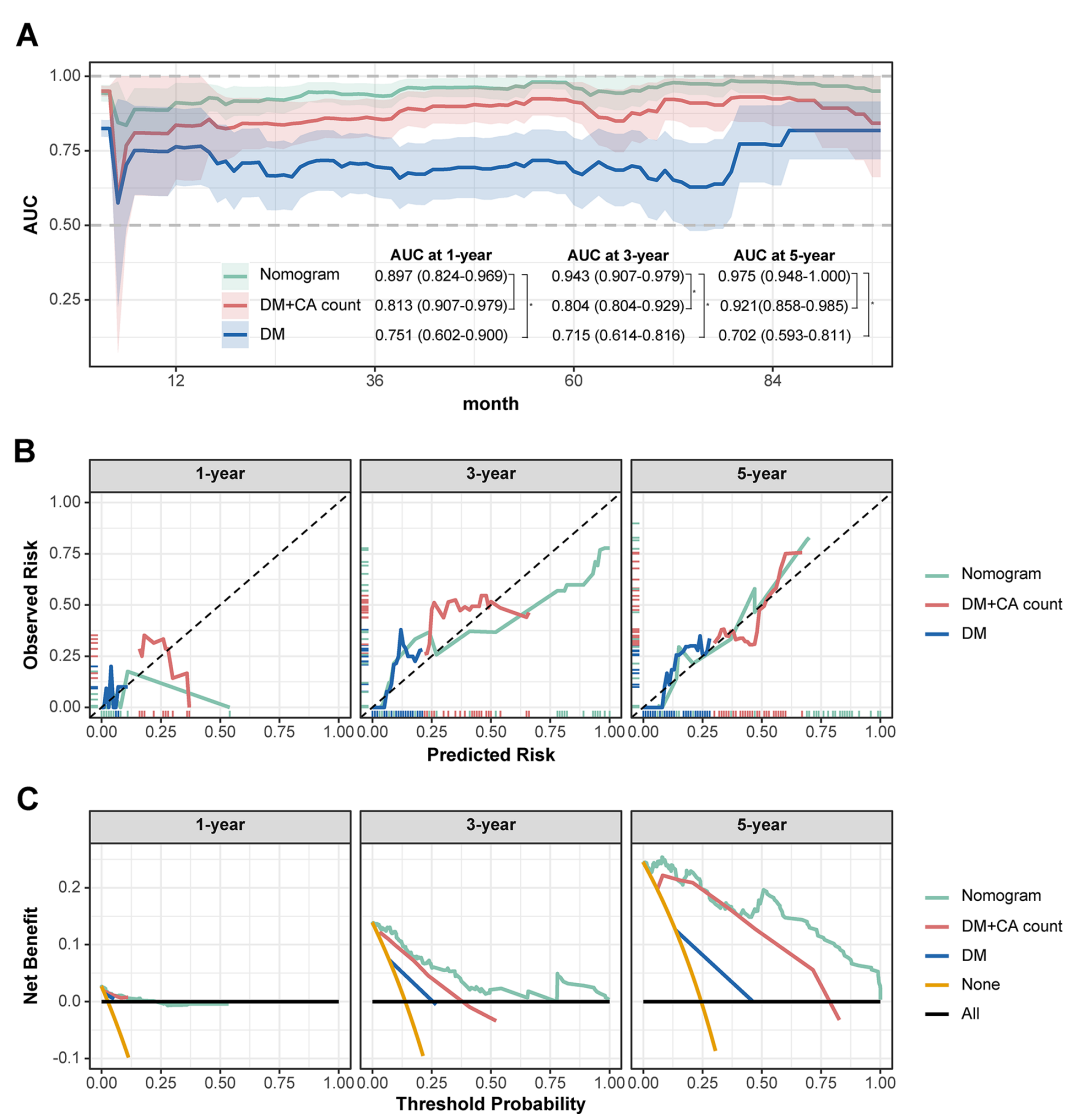
Time-dependent AUC values (with 95% confidence band) (**A**). Calibration plots (**B**) and decision curve (**C**) at 1-, 3- and 5-year. Nomogram, established by age, diabetes, calcification count, LDL-C, ALB and CHD/stroke. *DM* diabetes, *DM* diabetes, *CA-count* calcification count. * p < 0.05 by Delong test

We selected six target variables for the mortality risk prediction model—calcification count, age, CHD/stroke status, diabetes status, and LDL-C level (Supplementary file 3: Figure S2A, 2B)—via LASSO regression and cross-validation. No multicollinearity was found between variables (VIF of all valuables < 5, Supplementary file 3: Figure S2C, D). Based on the six indicators listed above, a nomogram and risk stratification model were created (Fig. 4A–D). The total point count of each patient increased with age, as did calcification, an increase in LDL-C, low albumin, comorbid diabetes, and CHD/stroke. Among these factors, ageing has the most significant impact on mortality. Collectively, the time-dependent AUC analysis demonstrated that the nomogram model consistently outperformed the DM+CA count and DM models, with AUC values improving over time: 0.897 at 1 year, 0.943 at 3 years, and 0.975 at 5 years (Fig. 5A). Significant differences were observed between the nomogram and DM models at all time points and between the nomogram and DM+CA model at 3 years (p < 0.05, Fig. 5A). The AUC of the nomogram tended to increase over time. Calibration plots showed that the nomogram model maintained alignment with the ideal diagonal line across 1 year, 3 years, and 5 years, indicating consistent performance (Fig. 5B). Decision curve analysis further revealed that the nomogram model provided the greatest net benefit across various threshold probabilities, with performance trends improving over time (Fig. 5C). Based on the model, we determined a cut-off of 94.12 (with a predicted probability of 2.33) for the total points, dividing the population into high-risk (25/40, 62.5%) and low-risk (17/245, 6.9%) groups. The high-risk group had a shorter survival time and lower survival rate in the same months (log-rank test p < 0.001, Fig. 4E). High-risk patients were 2.08 times more likely to die than low-risk patients (HR: 2.08, 95% CI: 1.78–2.43, p < 0.001, Fig. 4E, Supplementary file 1: Table S1).

## Discussion

In this study, ESRD patients in the early stages of HD with diabetes had lower blood phosphorus levels, calcium-phosphorus product levels, and serum ALB and iPTH levels than nondiabetic patients. Diabetes independently increases CVC risk. Furthermore, diabetes and multiple sites of calcification can shorten survival in the dialysis population. Older patients had lower serum albumin (ALB) levels and higher low-density lipoprotein cholesterol (LDL-C) levels, had diabetes, had several calcification sites, and had CHD/stroke had worse survival rates, which can better predict mortality.

Diabetes is an independent risk factor for cardiovascular death. Similarly, in patients with end-stage renal disease, cardiovascular disease is the leading cause of death, and the mediating factor of death is often severe cardiovascular calcification [4]. Several studies have shown that vascular calcification is unique in patients with diabetes and ESRD and that it usually differs from atherosclerosis in that it is mainly characterised by calcium-phosphorus deposits and hydroxyapatite crystals deposited in the intima-media of blood vessels, i.e., medial arterial calcification (MAC) [13]. A 10-year follow-up study as early as 1994 revealed that MAC is a vital independent risk factor for cardiovascular disease mortality with significantly stronger adverse effects than conventional atherosclerosis, especially in the diabetic population [14]. In this study, the incidence of coronary artery, abdominal aorta, heart valve, and overall calcification was significantly greater in diabetic patients than in nondiabetic patients. The survival curves of diabetic patients showed a steeper downward trend than those of nondiabetic patients. Similarly, VOLZKE H et al. [15] reported that among patients aged older than 45 years with a median survival age of 8.6 years, 21.6% of those with valvular calcification died, whereas only 6.5% of those without valve abnormalities died. Ming Li et al. [16] reported that patients with CVC had greater rates of all-cause and cardiovascular mortality than did those without CVC (50% vs. 14.8% and 25% vs. 7.0%, respectively, p < 0.05), suggesting that valvular calcification significantly increases the risk of all-cause and cardiovascular deaths, as well as new cardiovascular events, in patients undergoing HD.

Although the mechanisms underlying arterial calcification are multifaceted, most experts agree that the primary mechanism is the dedifferentiation or conversion of vascular smooth muscle cells (VSMCs) to an osteoblast/chondrocyte phenotype [17]. n turn, diabetes itself can trigger the emergence of this mechanism via various pathways. In diabetic patients’ arteries, membrane matrix proteins such as osteoblasts, collagen type I, and alkaline phosphatase are increased [18, 19], while in vitro, VSMCs in a high-glucose environment express more osteoclast transcription factors such as RUNX2, BMP-2, and osteocalcin, which promotes calcification. Elevated hyperglycaemia enhances the BMP-2/Msx2-Wnt pathway, causing some myofibroblasts to become osteogenic, while BMP-2 inhibition prevents arterial calcification [20]. Furthermore, osteoblastic differentiation of VSMCs can be induced by advanced glycation end products (AGEs), which accumulate in diabetes and promote vascular calcification through the AGE pathway [21]. These findings imply that the direct effects of hyperglycaemia, which in various ways induce VSMCs to resemble osteoblasts, are at least partially responsible for the increase in vascular calcification observed in diabetic individuals.

Additionally, age, calcium and phosphorus problems, dyslipidaemia, and hypoalbuminaemia increase vascular calcification and mortality. Age-related vascular pathology includes increased vascular calcification, generalisation of the *Windkessel* effect, and decreased tissue perfusion, which damages and disrupts end-target organs [22, 23]. Our research revealed that diabetic patients were older and had worse vascular calcification. Age also increased survival time by 12 points per decade according to the nomogram. In ageing patients, senescence, prelamin buildup, klotho insufficiency, oxidative stress, decreased autophagy, and microvessel production result in vascular calcification [23]. Calcium and phosphorus disorders contribute to vascular calcification. Previous research has revealed a favourable association between the degree of vascular calcification and hyperphosphatemia, particularly in HD patients [24–28]. Nevertheless, several investigations showed no link between phosphorus and vascular calcification in a meta-analysis [29]. In this study, diabetic patients were more prone to CVC but had lower levels of phosphorus and calcium-phosphorus products than nondiabetic patients, contradicting previous results. Similarly, Alexander et al. [30] reported that coronary artery calcification increased with decreasing serum phosphate and calcium phosphate levels. We hypothesise that individuals with diabetes may have a worse CVC, increased phosphorus excretion, and localised deposits at the HD entrance. For example, people with diabetes and nephropathy have higher eGFRs and greater calcium and phosphorus excretion [31]. Second, through the CML/RAGE axis, AGEs can facilitate apoptosis to release more matrix vesicles and establish a calcium–phosphorus deposition microenvironment [32–34]. Diabetic collagen deterioration may increase vascular wall calcium and phosphate accumulation [35]. Additionally, the clinical relevance of lipids in CVC is unknown. In CKD patients, elevated LDL-C does not cause coronary artery calcification [36]. A meta-analysis indicated that statins improve atherosclerotic disease but do not reduce coronary calcification [37]. Additionally, our study revealed that people with diabetes had significantly decreased serum ALB. Malnutrition is a risk factor for calcification and improper calcium and phosphorus metabolism [38]. Hence, supportive treatment is needed to improve nutritional status and serum ALB concentration.

Ultimately, a prediction model of mortality was preliminarily constructed for patients in the early stages of HD. Previously, Jürgen Floege et al. [39] developed a model for predicting the mortality of European HD patients; however, it did not account for diabetes and vascular calcification outcomes, limiting its applicability to Europe only. In this study, our model integrates diabetes, calcification, and other conventional indicators to enhance the prediction of patient mortality risk and to differentiate between high- and low-risk populations at the early stages of HD. Given China’s large population, unequal distribution of healthcare resources, rapid ageing, and substantial burden of cardiovascular and cerebrovascular events, there is a critical need for effective risk prediction models. This assessment facilitates prompt and targeted treatment for high-risk individuals, enabling clinicians to improve patient outcomes efficiently.

This study has the following innovations. *First,* the findings confirm the association between diabetes and vascular calcification in patients undergoing early HD, emphasising the importance of early intervention in diabetes patients. Intensive glycaemic control, including using gliclazide (modified release) and other hypoglycaemic agents, can reduce major macrovascular and microvascular events by 10%, primarily through a 21% reduction in nephropathy [40]. *Moreover*, our preliminary nomogram model can provide early intervention and therapeutic suggestions for vascular calcification avoidance in high-risk patients in China. Bone health must be prioritised for cardiovascular disease care, especially in Chronic Kidney Disease-Mineral and Bone Disorder. Calcimimetics, vitamin D analogues, and phosphate binders are used for this purpose. However, further research is needed to determine the best treatments [41].

This study has several limitations. First, this was a single-centre study, which may have resulted in insufficient clinical data and potential selection bias, thereby limiting the generalisability of the findings. Second, because it was observational, the study did not establish a causal relationship between diabetes, calcification, and all-cause mortality. Third, the sample size was relatively small, comprising only 285 patients with 42 deaths, which constrains the scope for multifactorial analysis and nomogram model development. This limitation may lead to results skewed by extreme values; thus, the model cannot be fully validated and should be considered a preliminary reference. Fourth, the study only evaluated valves, thoracic aorta, and coronary arteries, potentially overlooking calcifications in other anatomical locations. Finally, several other factors may still influence the outcomes, such as patients’ left ventricular ejection fraction, glucose levels, hypoglycaemic drug therapy during follow-up, environment, family history, and behavioural characteristics. Further research with a more comprehensive dataset is required to address these limitations and enhance the model’s robustness.

## Conclusion

We conclude that diabetes is an independent risk factor for vascular calcification in patients with ESRD who are in the early stages of hemodialysis and that age and severe calcification are independent predictors of all-cause mortality. Additionally, it is imperative to develop and validate more accurate models for predicting patient risk to enhance clinical practice and improve patient outcomes.

### Supplementary Information

Supplementary file 1: Table S1.

Supplementary file 2: Figure S1.

Supplementary file 3: Figure S2.

## Data Availability

The datasets analyzed during the current study are not publicly available due to patient privacy but are available from the corresponding author on reasonable request.

## Abbreviations

DM: Diabetes
HD: Hemodialysis
CA: Calcification
CVC: Cardiovascular calcification
CKD: Chronic kidney disease
CHD: Coronary heart disease
BMI: Body mass index
ALB: Albumin
HbA1c: Blood glycosylated hemoglobin
Pi: Phosphate
Ca: Corrected by albumin
TC: Total cholesterol
TG: Triglyceride
LDL-C: Low-density lipoprotein cholesterol
HDL: High-density lipoprotein cholesterol
eGFR: Estimate glomerular filtration rate
VIF: Variance inflation factor
AGEs: Advanced glycation end-products
CMH: Cochran–Mantel–Haenszel
ROC: Receiver operating characteristic
AUC: Area under the curve
