# Supplementary material for: Association of diabetes with cardiovascular calcification and all-cause mortality in end-stage renal disease in the early stages of hemodialysis: a retrospective cohort study
Source: Cardiovasc Diabetol. 2024 Jul 18;23:259. doi: 10.1186/s12933-024-02318-8 (PMC11264609; doi:10.1186/s12933-024-02318-8)
Supplement: Supplementary file 1 — Table S1. [file 12933_2024_2318_MOESM1_ESM.docx]

**Table S1. Risk of all-cause mortality by low-high stratification risk group**

|  | | **Mortality (%)** | **HR (95% CI)** | ***p-value*** |
| --- | --- | --- | --- | --- |
| Total points (per 10 units) | | 42/285 (14.7) | 2.08 (1.78-2.43) | <0.001 |
| Risk stratification | |  | | |
|  | Low risk (<94.12 points) | 17/245 (6.9) | Ref | - |
|  | High risk (≥94.12 points) | 25/40 (62.5) | 26.89 (12.63-57.23) | <0.001 |

HR hazard ratio, CI confidence interval
