# Supplementary material for: Association of diabetes with cardiovascular calcification and all-cause mortality in end-stage renal disease in the early stages of hemodialysis: a retrospective cohort study
Source: Cardiovasc Diabetol. 2024 Jul 18;23:259. doi: 10.1186/s12933-024-02318-8 (PMC11264609; doi:10.1186/s12933-024-02318-8)
Supplement: Supplementary file 2 — Figure S1. [file 12933_2024_2318_MOESM2_ESM.pdf]

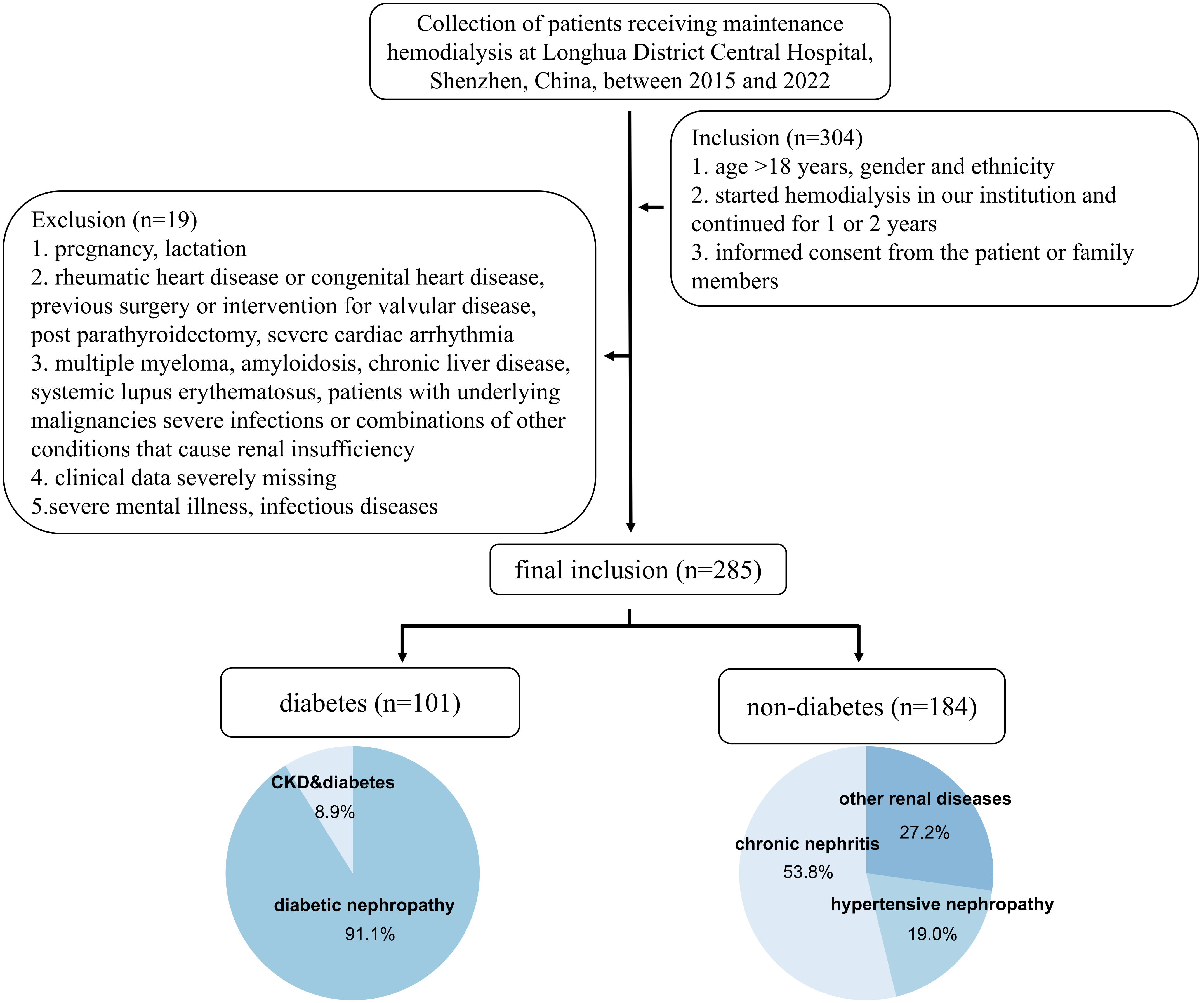
Collection of patients receiving maintenance hemodialysis at Longhua District Central Hospital, Shenzhen, China, between 2015 and 2022
Inclusion (n=304)
1. age >18 years, gender and ethnicity
2. started hemodialysis in our institution and continued for 1 or 2 years
3. informed consent from the patient or family members
Exclusion (n=19)
1. pregnancy, lactation
2. rheumatic heart disease or congenital heart disease, previous surgery or intervention for valvular disease, post parathyroidectomy, severe cardiac arrhythmia
3. multiple myeloma, amyloidosis, chronic liver disease, systemic lupus erythematosus, patients with underlying malignancies severe infections or combinations of other conditions that cause renal insufficiency
4. clinical data severely missing
5.severe mental illness, infectious diseases
final inclusion (n=285)
diabetes (n=101)
non-diabetes (n=184)
CKD&diabetes
8.9%
diabetic nephropathy
91.1%
other renal diseases
27.2%
chronic nephritis
53.8%
hypertensive nephropathy
19.0%
