# Supplementary figures and images for: Association of diabetes with cardiovascular calcification and all-cause mortality in end-stage renal disease in the early stages of hemodialysis: a retrospective cohort study
Source: Cardiovasc Diabetol. 2024 Jul 18;23:259. doi: 10.1186/s12933-024-02318-8 (PMC11264609; doi:10.1186/s12933-024-02318-8)

A

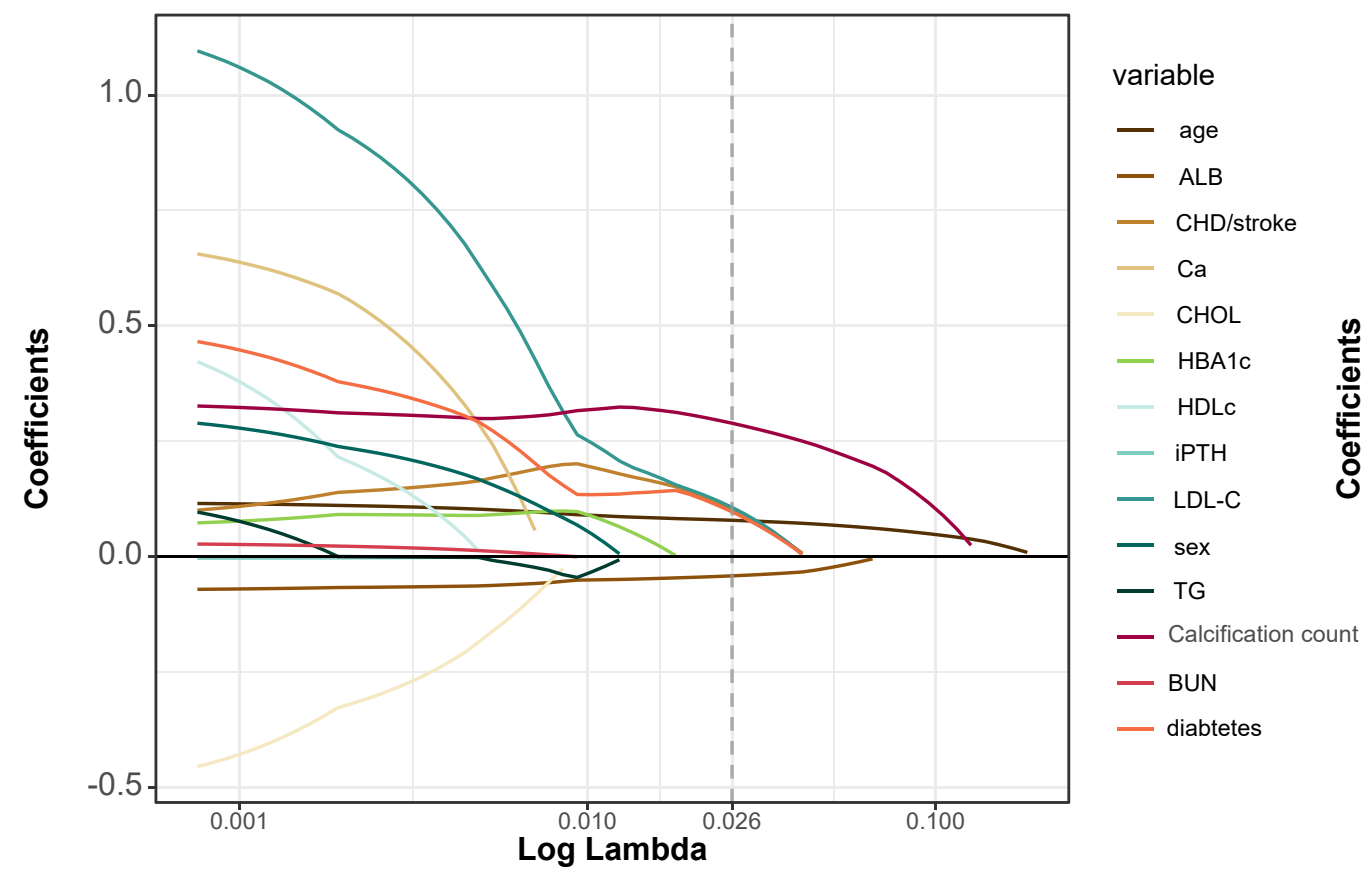

B

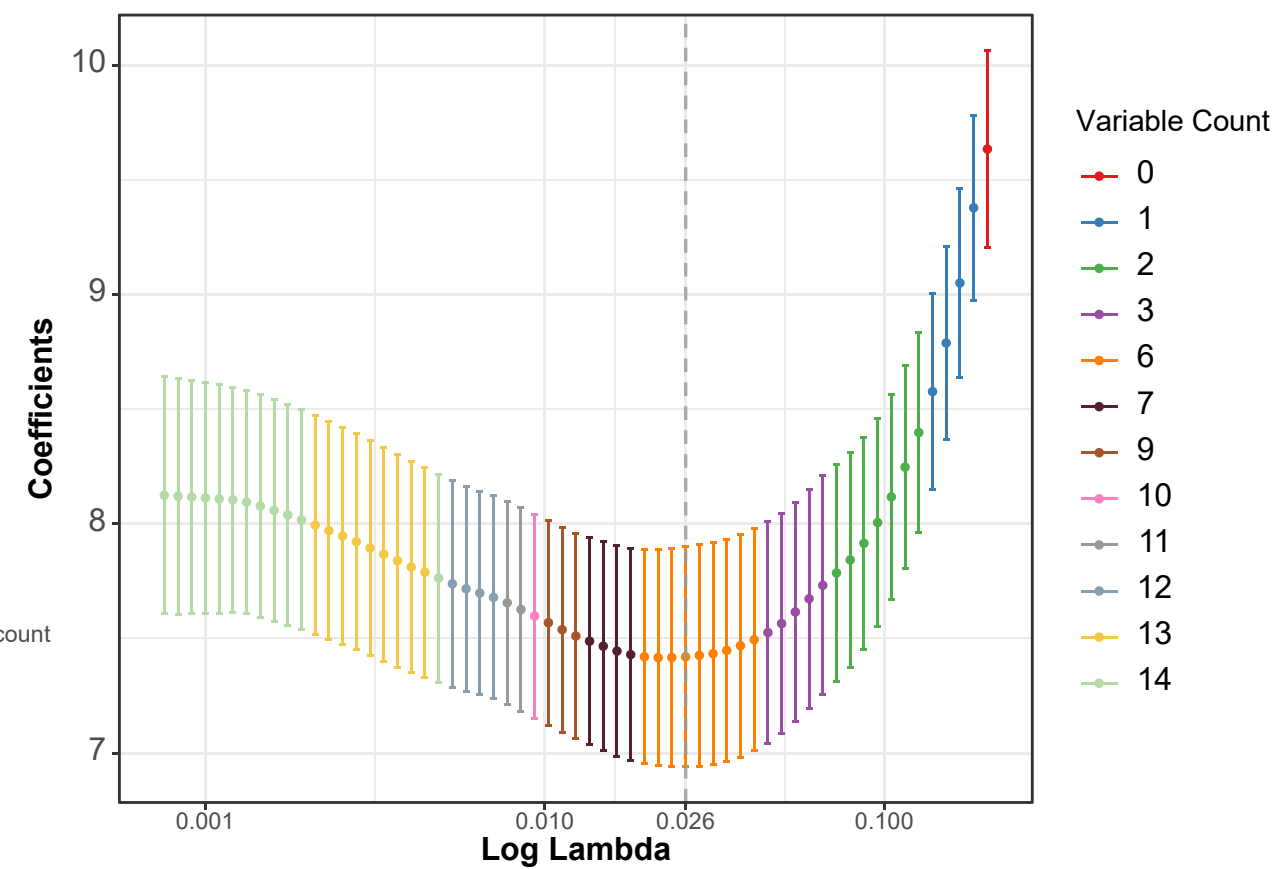

C

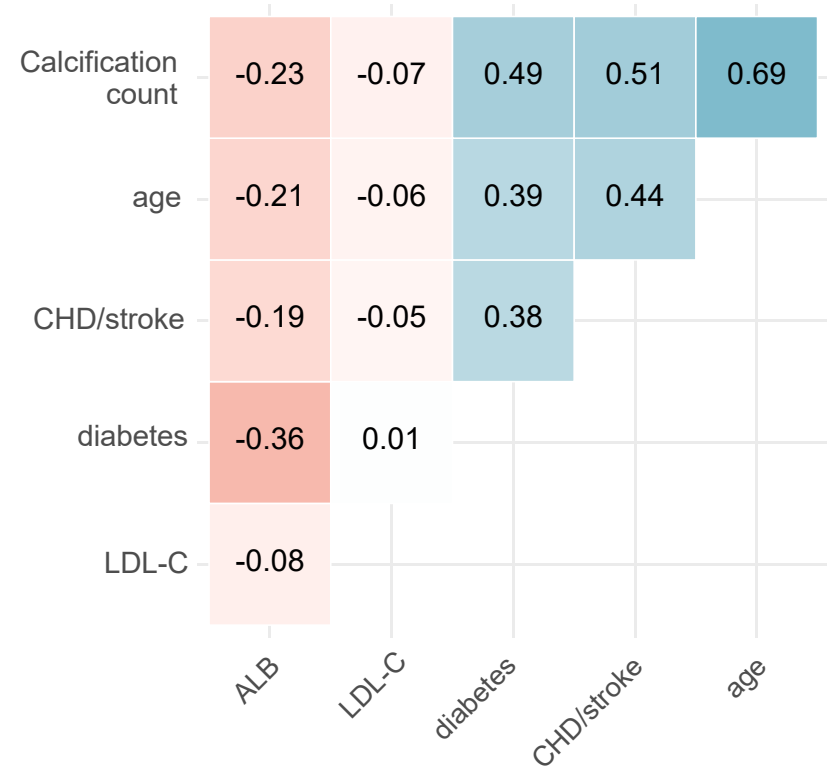

D

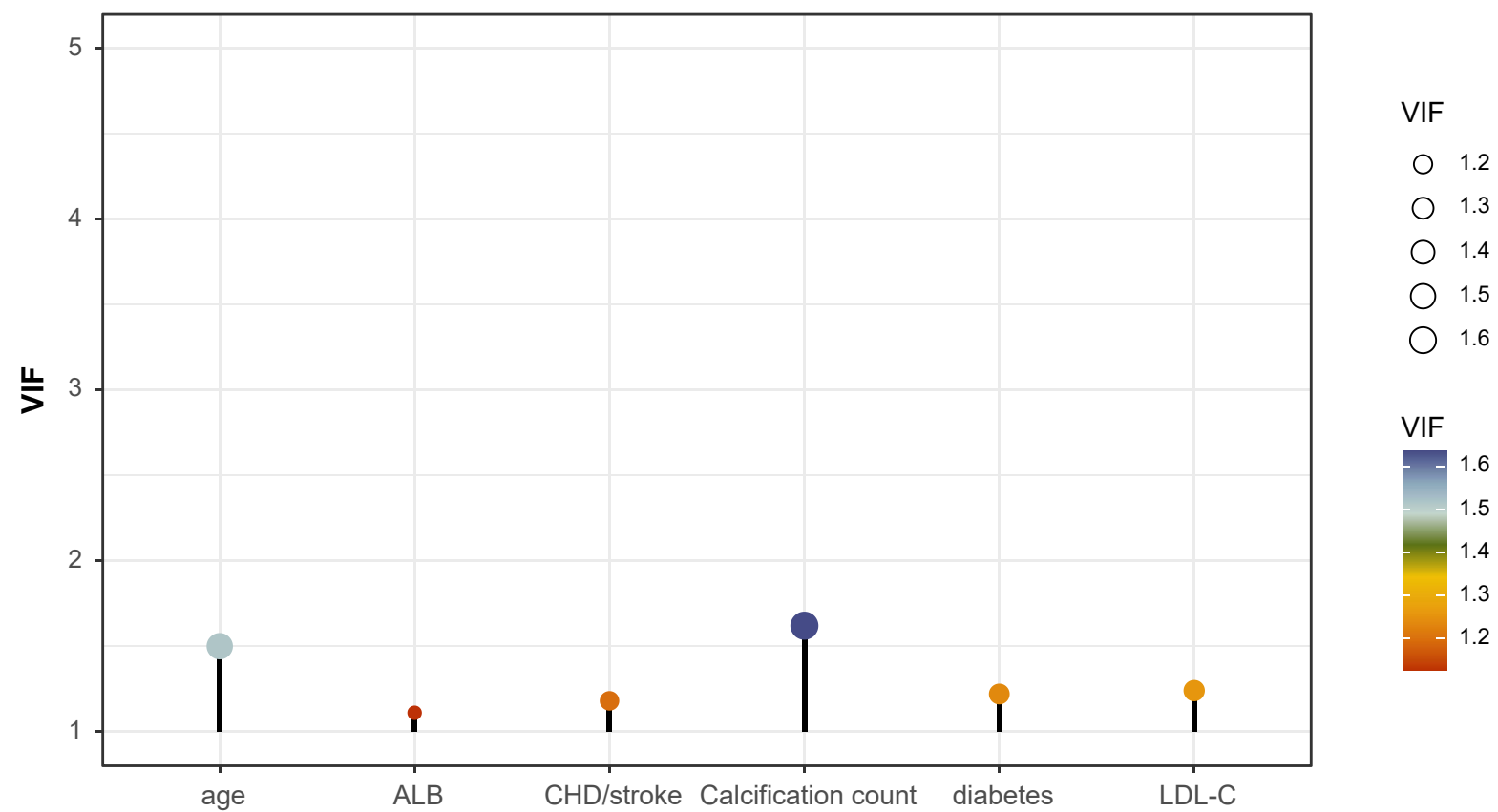

Supplement: Supplementary file 3 — Figure S2. [file 12933_2024_2318_MOESM3_ESM.pdf]
